# Carbonate Ion-Enriched Hot Spring Water Promotes Skin Wound Healing in Nude Rats

**DOI:** 10.1371/journal.pone.0117106

**Published:** 2015-02-11

**Authors:** Jingyan Liang, Dedong Kang, Yingge Wang, Ying Yu, Jianglin Fan, En Takashi

**Affiliations:** 1 School of Medicine, Yangzhou University, Yangzhou, China; 2 Basic Medicine and Nosography, Nagano College of Nursing, Komagane, Japan; 3 Department of Molecular Pathology, Interdisciplinary Graduate School of Medicine and Engineering, University of Yamanashi, Yamanashi, Japan; Hamamatsu University School of Medicine, JAPAN

## Abstract

Hot spring or hot spa bathing (*Onsen*) is a traditional therapy for the treatment of certain ailments. There is a common belief that hot spring bathing has therapeutic effects for wound healing, yet the underlying molecular mechanisms remain unclear. To examine this hypothesis, we investigated the effects of Nagano hot spring water (rich in carbonate ion, 42°C) on the healing process of the skin using a nude rat skin wound model. We found that hot spring bathing led to an enhanced healing speed compared to both the unbathed and hot-water (42°C) control groups. Histologically, the hot spring water group showed increased vessel density and reduced inflammatory cells in the granulation tissue of the wound area. Real-time RT-PCR analysis along with zymography revealed that the wound area of the hot spring water group exhibited a higher expression of matrix metalloproteinases-2 and -9 compared to the two other control groups. Furthermore, we found that the enhanced wound healing process induced by the carbonate ion-enriched hot spring water was mediated by thermal insulation and moisture maintenance. Our results provide the evidence that carbonate ion-enriched hot spring water is beneficial for the treatment of skin wounds.

## Introduction

Balneotherapy, a water immersion therapeutic method for treatment of many types of diseases, is a traditional therapy practiced by many countries around the world[1–3]. In Japan, hot springs are popular in almost all regions of the country, due to the high density of active volcanoes[4]. There has been a long history of Japanese people bathing in hot springs for both sanitation and enjoyment. There have been many reports that have shown that hot spring water may have some therapeutic effects for the treatment of various diseases, such as cardiovascular disease[5–8], atopic dermatitis[9,10], ankylosing spondylitis[11], asthma[12], inflammatory arthritis[13], rheumatic disease[14], and rhinosinusitis[15]. It is widely recognized that a tranquil soak in a hot spring provides relief from the pain of stressed muscles and tired joints. For example, in Japan’s Warring States Period (1493–1590), hot spas were used for treating wounded soldiers[16]. Despite this long history and tradition, the pathological mechanisms for the therapeutic effects of hot springs are still not fully understood. In this regard, we were interested in the therapeutic effects of carbonate ion-enriched hot spring water on skin wound healing. For this undertaking, we established a skin wound model on the superior part of the posterior extremity of nude rats, and compared the effects of Nagano hot spring water with hot water or no treatment on the skin healing process. The results of our study indicated that carbonated hot spring water therapy improves wound healing compared with hot water at the same temperature.

## Materials and Methods

### Animals

Thirty-four healthy male Wistar Yagi nude rats at eight weeks (HWY/Slc, SLC, Inc. Shizuoka, Japan) were used in this study. The rats were randomly divided into three groups: untreated control (n = 12), hot water control (n = 10), and hot spring water (n = 12) groups. All animals were fed a standard chow diet with free access to water. This study was carried out in strict accordance with the recommendations in the Guide for the Care and Use of Laboratory Animals of the National Institutes of Health. The protocol was approved by the Committee on the Ethics of Animal Experiments of the Nagano College of Nursing. All surgery was performed under ketamine hydrochloride/medetomidine chloride sodium anesthesia, and all efforts were made to minimize suffering.

### Experimental design

Rats were anesthetized by intraperitoneal injection of ketamine hydrochloride (75 mg/kg Daiichi Sankyo, Tokyo, Japan) and medetomidine chloride (0.5 mg/kg Zenoaq, Fukushima, Japan). To make a skin wound model, two round wounds (approximately 10 mm in diameter) were punched into each side of the superior region of the posterior extremity. The wounds were kept open, and the rats were housed individually. Three days later, all rats were soaked in either hot spring water or hot water of the same temperature (42°C), for fifteen minutes every three days for eight weeks. The temperature (42°C) was selected because our pioneer study showed that among three hot springs with 38, 40, and 42°C, 42°C hot spring was more effective than other two in terms of skin ulcer healing speed (data not shown). In addition, hot spring with 42°C is one of the typical temperatures used in Japan[17]. The hot spring water was collected from Minowa Spring, Nagano, and its components are shown in Table 1.

**Table 1:** Analysis of Nagano spring ingredients.

Nature of spring	Sodium bicarbonate spring
Sodium ion (mg/L)	565.4
Calcium ion (mgL)	5.6
Chlorine ion (mg/L)	62
Carbonate ion (mg/L)	1375.3
pH value	8.01
Mass of dissolved ingredients	2057.6

### Pathological analysis

The gross area of the skin wound was photographed by a digital camera AM-313 (Sato Shoji Co., Kawasaki, Japan) every week and measured with an image analysis software, NIH Image J ver.1.47 (National Institutes of Health, Bethesda, MD <http://imagej.nih.gov/ij/download.html>). The initial relative open wound area was defined as 100%, and later wound area data (relative area of wound to initial wound) were calculated using macroscopic photographs at the indicated time points[18]. At the end of the experiment, the rats were sacrificed by injection of an overdose of sodium pentobarbital solution. For histological examination, the skin wound specimens were fixed in 10% buffered formalin, embedded in paraffin, and sections (3 μm thick) were stained with hematoxylin and eosin (HE) staining, and Masson's trichrome (MT) stain. To analyze blood vessel formation of the wound, we used HE-stained sections and evaluated the angiogenesis index by counting the number of blood vessels under a light microscope. The vascular count from six random high-magnification fields was calculated and expressed as the number of vessels per mm^2^. To evaluate macrophage infiltration, the paraffin sections were immunohistochemically stained with monoclonal antibody against rat macrophages (RM-4, working concentration 10 μg/ml) (TransGenic Inc., Kobe, Japan) using the method described previously[19]. Macrophages stained by RM-4 antibody were calculated from five high-power fields (HPF) and expressed as mean number/HPF. Wound healing and vessel density were observed and quantitated by Lumina Vision V2.2 image analysis software (Mitani Co., Tokyo, Japan).

### MMP expression in the skin wound

Total RNA was extracted from the skin wounds using Trizol solution, and cDNA was prepared with a QuantiTect reverse transcription kit (QIAGEN Inc., Valencia, CA) according to previous methods[19]. The expression of matrix metalloproteinase (MMP)-2 and-9, along with GAPDH (as an internal control) was analyzed by real-time RT-PCR. Sequences of the primers were as follows: for MMP-2 5’-GGAAGCATCAAATCGGACTG-3’ (forward), 5’-TAACCAGGCCTCTTCACGTC-3’ (reverse); for MMP-9 5’-AACTCGGCAGGAGAGATGTG-3’ (forward), 5’-CACTTCTTGTCAGCGTCGAA-3’ (reverse). Enzymatic activity of MMPs was also evaluated by zymography using gelatin as a substrate[20].

Measurement of surface temperature and moisture

The rats were bathed in either hot water or hot spring water for 5, 10 and 20 minutes. The surface temperature of the rat’s skin was then immediately measured by the FLIR i3 infrared ray thermography non-contact thermometer (FLIR Systems, Inc. Japan), and moisture content was measured by the DM-R2 skin moisture meter (Panasonic Co. Tokyo, Japan).

### Statistical analysis

All values were expressed as mean ± SD. The student’s *t* test (two datasets), one-way ANOVA with Tukey’s multiple comparisons test, or the Kruskal-Wallis test (three datasets) were used for parametric or nonparametric analysis with IBM SPSS Statistics 21 software. In all cases, the statistical significance was set at *p*<0.05.

## Results

### Macroscopic and histological evaluation of skin wounds

**Fig 1:**
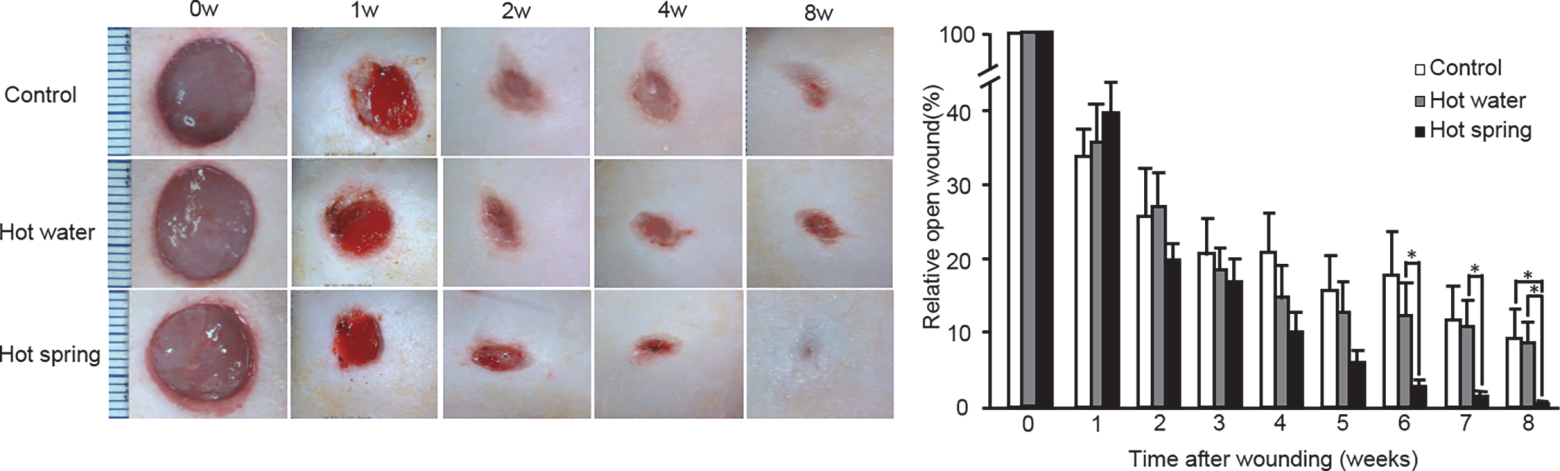
Representative pictures of a skin wound at different time points (left) and quantitation of wound healing (right). The wound closure was quantitated as described in the Materials and Methods section. Data are expressed as the mean ± SD. n = 12, 10, and 12 for the untreated control, hot water control, and hot spring water groups, respectively. *P < 0.05.

As shown in Fig. 1, hot spring water led to the enhancement of skin wound healing compared with the other two groups. The wound area (expressed as the relative open wound area[18]) was smaller in the hot spring water group than the other two groups after four weeks, and statistical significance was attained at six weeks. At eight weeks, the wounds of the hot spring water group were almost completely healed.

**Fig 2:**
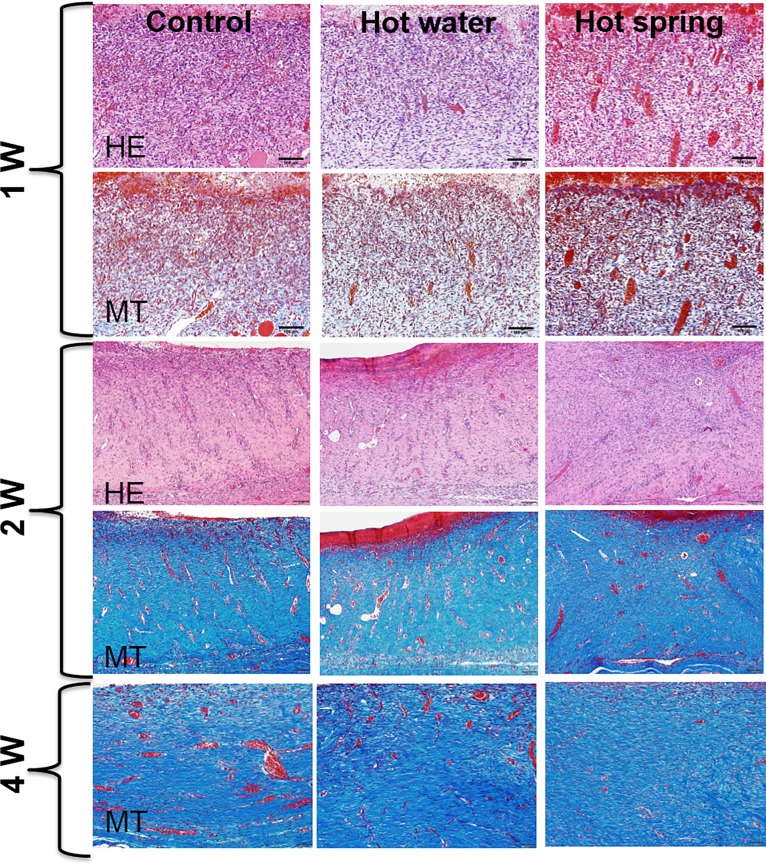
Histological evaluation of the skin wound. Representative micrographs of a skin wound stained by either hematoxylin and eosin (HE) or Masson trichrome (MT) stain.

**Fig 3:**
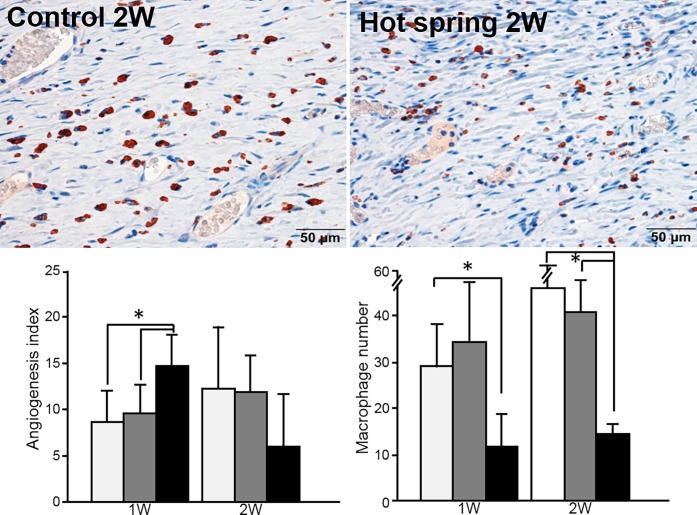
Immnohistochemical staining of macrophages and morphometric evaluation of the skin wound vessels and macrophages. Micrographs of macrophages immunohistochemically stained by RM-4 antibody (top panel). The angiogenesis index and macrophage number were quantified (bottom panel). Data are expressed as the mean ± SD. n = 6 for each group for angiogenesis analysis. n = 3 for macrophage calculation. *P <0.05.

Under light microscopy, the wounds of the hot spring water group were characterized by an increased number of capillary vessels in the granulation tissue after one week compared with the other two groups (Figs. 2–3). These capillary vessels were then rapidly replaced by increased fibrosis after two weeks, and the wound was totally replaced by fibrosis after four weeks. To evaluate the macrophage number in the wound area, we performed immunohistochemical staining and calculated macrophages. As shown in Fig. 3, macrophages were significantly reduced in the hot spring water group.

### MMP-2 and MMP-9 expression in the wounds

**Fig 4:**
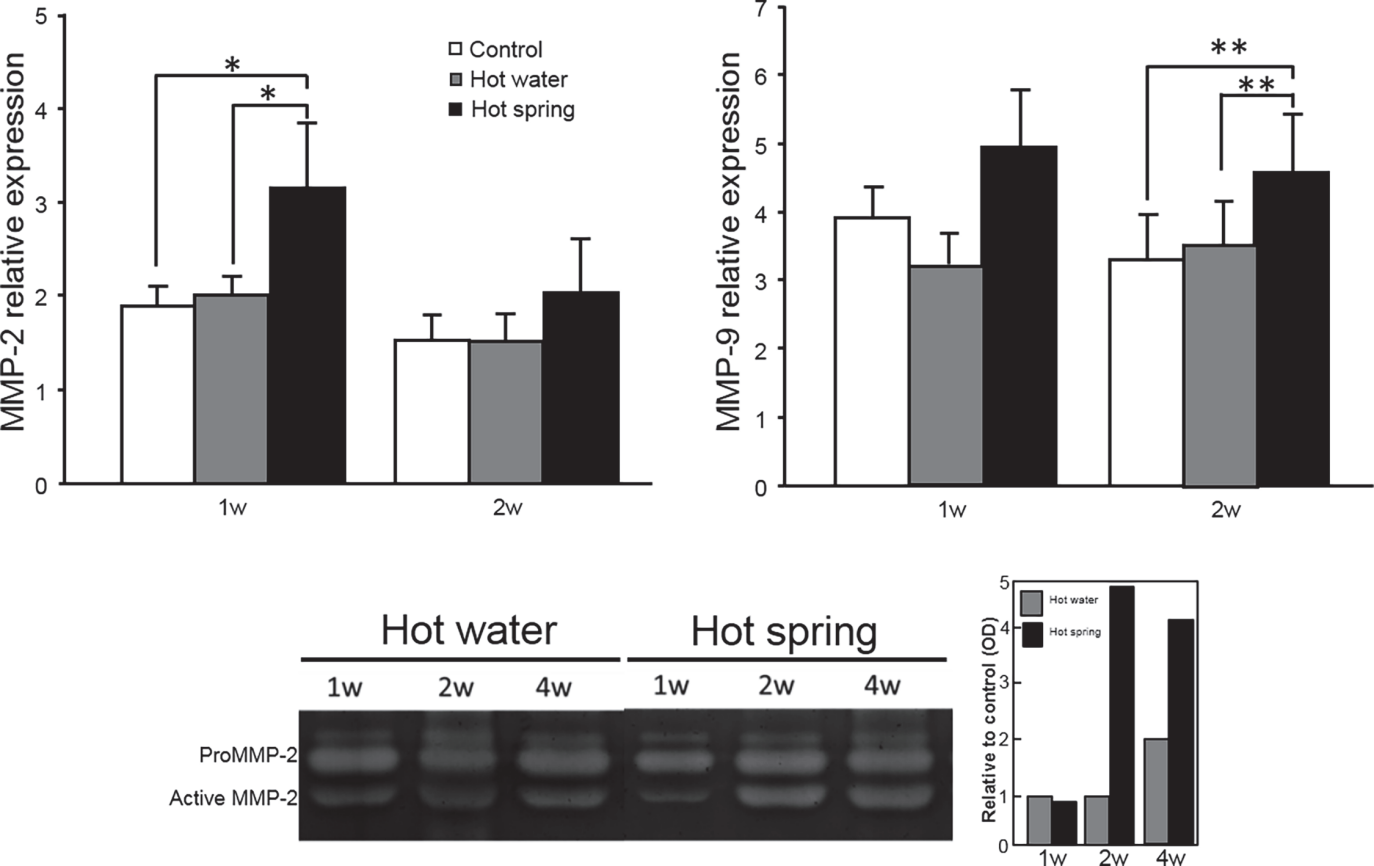
Expression of MMP-2 and MMP-9 and gelatin zymography in skin wounds. mRNA expression of MMP-2 and MMP-9 was quantified using real-time RT-PCR (top panel). Data are expressed as the mean ± SD. n = 5 for each group. * *P* <0.05 and ** P<0.01. Gelatin zymography was performed as described in the Materials and Methods (bottom panel). Representative data of hot water and hot spring water groups was shown and the signals of pro-MMP-2 and active MMP-2 was scanned by a densitometry and expressed as optical density (OD) relative to the hot water at 1 week (bottom right).

To explore the possible molecular mechanisms responsible for the enhanced wound healing induced by the hot spring water, we investigated the expression of MMP-2 and MMP-9 mRNA, two important regulators in maintaining the extracellular matrix, angiogenesis, and tissue repair. As shown in Fig. 4, the wounds of the hot spring water group showed higher expression of MMP-2 after one week, and MMP-9 after two weeks compared to the other two groups. In support of this mRNA expression, gelatin zymography also showed that MMP-2 activity was increased in the hot spring group (Fig. 4) while MMP-9 was not remarkable (data not shown).

### Surface temperature and moisture change in the skin

**Fig 5:**
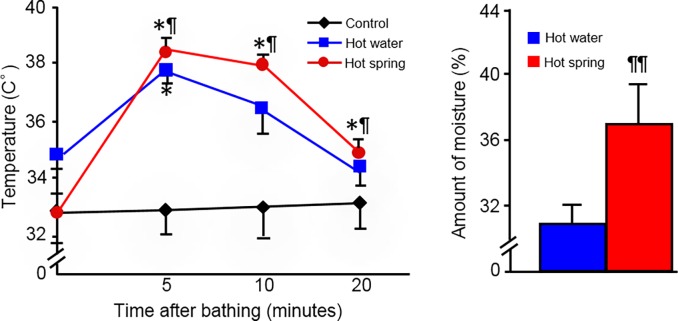
Analysis of temperature and moisture. A thermogram shows temperature changes on the skin after bathing for 5, 10 and 20 minutes. Moisture changes of the skin surface are compared between the hot water and hot spring water groups. Data are expressed as the mean ± SD. n = 5 for each group. * or ** *P* <0.05 or <0.01 vs untreated control; ^¶^ *P*<0.05 vs the hot water group.

Because the wound healing process was faster in the hot spring water group compared with the other two control groups, temperature may not be critical for the beneficial effects provided by hot spring water. We further compared the surface temperature and moisture changes of the skin after bathing in either hot spring water or hot water for 5, 10 and 20 minutes. As shown in Fig. 5, hot spring water led to an increased temperature of the skin much more quickly than the hot water group, and also increased the moisture content of the skin.

## Discussion

Chronic skin ulcers can be caused by pressure, venous stasis, or diabetes mellitus[21]. Intractable skin ulcers and decubitus are also a common and serious problem met in clinical nursing facilities. Unfortunately, there are no effective treatments for these skin complications. We investigated whether it was possible to use hot spring as an alternative treatment for intractable skin ulcers by using a nude rat skin ulcer model. In the present study, we demonstrated that carbonate ion-enriched hot spring water (from Nagano) enhanced the skin wound healing process in nude rats compared with those either untreated or treated with just hot water. Microscopically, the wound area treated by hot spring water was characterized by increased angiogenesis and reduced macrophages in the early stage after injury and quick accumulation of collagen in the later stage. Furthermore, mRNA expression of MMP-2 and MMP-9, two important regulators of the healing process, was increased in the wound lesions treated by hot spring water.

Skin wound healing is a complex process that can be divided into at least three continuous and overlapping processes: inflammatory reaction, a proliferative process leading to tissue restoration, and eventually, tissue remodeling[22].

Several possible mechanisms have been proposed to explain the therapeutic effect of hot spring water on skin wound healing. For example, an increased temperature can increase blood flow and improve circulation both locally and systemically[23,24]. The components of the hot spring water are also thought to have an antimicrobial effect directly on the skin [9], and may also penetrate the skin to act systemically.

Although the thermal effect has been thought to play the major role in the enhancement of skin wound healing after hot spring water treatment, there are other factors that may be more critical. In the present study, we found that hot spring water treatment increased skin healing more quickly than just hot water of the same temperature. This suggests that the components of the hot spring water may have more of an important effect than temperature alone. To explore this possibility, we performed a preliminary study using artificial hot spring water, which consisted of roughly the same components as natural hot spring water, and found that it was also effective (Liang et al., unpublished observations). Investigation of which component(s) in the hot spring water are required for a therapeutic effect should be investigated in the future. It is worth noting that the surface temperature and moisture change in the rat skin was prominently enhanced after treatment with hot carbonate ion-enriched spring water compared to the hot water control of the same temperature. These data suggest that immersion in carbonate ion-enriched water may improve thermal insulation and preservation of moisture. Nevertheless, the molecular mechanism underlying beneficial effects of carbonate ion-enriched spring water is currently unknown and it will be interesting to elucidate this issue in future. The current study suggests that the hot spring exerts anti-inflammatory effects because macrophage infiltration was reduced. Since many growth factors are involved in wound repair and regeneration[25], it may be quite difficult to elucidate which factors are involved and affected by bathing in hot spring water. Recently, cathepsins have shown to play an important role in angiogenesis[26,27] and again it opens a possibility that whether these molecules may be involved in the healing process mediated by hot spring. In spite of this, we focused on the expression of MMP-2 and MMP-9, two important gelatinases (gelatinase A and gelatinase B) which are capable of degrading extracellular matrix, gelatin secreted by various cells such as macrophages, endothelial cells and fibroblasts. These two MMPs play important roles in wound healing processes including keratinocyte migration, granulation tissue remodeling[28] and angiogenesis[29].

As shown in the present study, hot spring water group promoted blood vessel formation and inhibited macrophage infiltration in granulation tissue in rat skin after one week. It is quite likely that increased MMP-2 and MMP-9 may be derived from fibroblasts rather than macrophages. Increased MMP-2 and MMP-9 may facilitate the removal of extracellular matrices and thus allow both endothelial cell and keratinocyte migration, by which the healing process is enhanced.

In the present study, we used rat models to evaluate the therapeutic effects of the hot spring. However, it should be pointed out that the results obtained may be limited because it is not known whether hot spring causes other responses (such as stress) in rats, which may be different from humans.

In conclusion, bathing in carbonate ion-enriched hot spring water enhanced skin wound healing in nude rats. Hot spring bathing may inhibit inflammatory reactions and increase healing process. These results indicate that the therapeutic effect of hot spring may be useful for the treatment of skin ulcers.
